# *In Silico* Analysis of Antibiotic Resistance Genes in the Gut Microflora of Individuals from Diverse Geographies and Age-Groups

**DOI:** 10.1371/journal.pone.0083823

**Published:** 2013-12-31

**Authors:** Tarini Shankar Ghosh, Sourav Sen Gupta, Gopinath Balakrish Nair, Sharmila S. Mande

**Affiliations:** 1 BioSciences R&D Division, TCS Innovation Labs, Tata Consultancy Services Ltd., Pune, Maharashtra, India; 2 Translational Health Sciences and Technology Institute, Gurgaon, Haryana, India; University of Hyderabad, India

## Abstract

The spread of antibiotic resistance, originating from the rampant and unrestrictive use of antibiotics in humans and livestock over the past few decades has emerged as a global health problem. This problem has been further compounded by recent reports implicating the gut microbial communities to act as reservoirs of antibiotic resistance. We have profiled the presence of probable antibiotic resistance genes in the gut flora of 275 individuals from eight different nationalities. For this purpose, available metagenomic data sets corresponding to 275 gut microbiomes were analyzed. Sequence similarity searches of the genomic fragments constituting each of these metagenomes were performed against genes conferring resistance to around 240 antibiotics. Potential antibiotic resistance genes conferring resistance against 53 different antibiotics were detected in the human gut microflora analysed in this study. In addition to several geography/country-specific patterns, four distinct clusters of gut microbiomes, referred to as ‘Resistotypes’, exhibiting similarities in their antibiotic resistance profiles, were identified. Groups of antibiotics having similarities in their resistance patterns within each of these clusters were also detected. Apart from this, mobile multi-drug resistance gene operons were detected in certain gut microbiomes. The study highlighted an alarmingly high abundance of antibiotic resistance genes in two infant gut microbiomes. The results obtained in the present study presents a holistic ‘big picture’ on the spectra of antibiotic resistance within our gut microbiota across different geographies. Such insights may help in implementation of new regulations and stringency on the existing ones.

## Introduction

The dissemination of antibiotic resistance is a global health problem, with many countries already implementing several regulations for the restrictive use of antibiotics [1]. The rampant and unrestrictive use of antibiotics (especially in insufficient dosage without prescription) over the past few decades has resulted in infectious bacteria having remarkably evolved their genomes to ensure survival by counter-acting the action of almost all known antibiotics [2], [3]. These survival strategies include mutations of the antibiotic target, acquisition of genes facilitating the efflux or breakdown of the antibiotic, and even alternate pathways to ‘evade’ the action of the antibiotics [4]. Furthermore, bacteria have successfully devised mechanisms to transfer these resistance properties to a broad range of other unrelated bacteria [5].

The gut harbors the largest microbial communities in the human body. Recent studies on gut microbiota of individuals have also revealed that commensal gut microflora can act as reservoirs of antibiotic resistance [6]. These bacteria have the potential to transfer antibiotic resistance genes to transient allochthonous and potentially pathogenic bacteria, thereby helping them increase their chances of survival and consequently their duration of persistence without the selective antibiotic pressure [7], [8]. This paves the way for pathogenic bacteria to further invade and colonize the human gut and aggravating otherwise uncomplicated clinical situations to potentially life threatening ones. The above problem is further exacerbated by increasing people-to-people contact around the world, wherein antibiotic resistant bacteria originating in one corner of the globe are quickly transferred and disseminated in distantly separated countries at large geographic separations [9]. Livestock and aquaculture are other areas where antibiotics are routinely used (predominantly in sub-therapeutic dosages) either for growth promotion therapy or prophylactic reasons contributing further to the spread of resistance. According to estimates in the US, quantities of antibiotics used in live stock production range from 18 million to 25 million pounds [10].

It is not only important to characterize the overall extent and spread of antibiotic resistance in individuals belonging to diverse geographic locations, but also identify the geography/age specific patterns in antibiotic resistance across various populations. Comprehensive assessment of the overall abundance and country-specific patterns in antibiotic resistance is likely to provide valuable insights. These insights may help government agencies in implementing new regulations on the restrictive use of antibiotics, besides increasing the stringency of existing ones. In addition, results of such study may also help in designing country-specific strategies for the development of newer antibiotics.

Previous studies have estimated that more than 99% of microbes residing in diverse environments cannot be cultured using the traditional laboratory based techniques [11]. Consequently, the applicability of traditional approaches for detection and characterization of antibiotic resistance genes present in hitherto unknown organisms in the gut microflora of individuals is expected to be limited. The metagenomics approach bypasses the above limitation by facilitating the direct extraction, sequencing and analysis of the genomic content (referred to as the metagenome) of entire community of microbes residing in a given environment. Researchers can now profile and study the taxonomic and functional properties of known as well as hitherto undiscovered microbial organisms residing in any environment. The metagenomics approach has enabled researchers to understand and unravel the role of the gut microbial community, referred to as the microbiome, in several physiological disorders and diseases, e.g, obesity, Crohn’s disease, cirrhosis and diabetes. Several studies have also adopted the metagenomics based functional screening approach to profile the antibiotic resistance in clone libraries of microbial genomic fragments extracted from the gut of a group individuals belonging to specific localities [12]–[14]. One of these studies has attempted to detect the presence of specific antibiotic resistance genes using PCR based amplification in the clone libraries corresponding to the different metagenomes [14]. Other studies have focused on the detection of clones conferring resistance against a small subset of antibiotics like tetracycline, and other beta-lactam based antibiotics like penicillin and ampicillin [13]. However, the current picture of the pattern of abundance of antibiotic resistance in the gut environment of individuals is limited with respect to the sample size of individual populations, the number of antibiotics as well as the different geographical locations being profiled.

The Antibiotic Resistance Genes Database (ARDB) contains a comprehensive repository of around 23,078 antibiotic resistance gene/protein sequences conferring resistance against more than 240 known antibiotics [15]. In addition, several gut metagenomic data sets from individuals of diverse geographies and nationalities have been reported in literature [16]–[19]. Thus the availability of the gut metagenomes as well as a database like ARDB provides a unique storehouse of information that can be mined for the presence of antibiotic resistance genes in individuals from various geographies. Two recent studies have indicated correlations between antibiotic resistance profiles and country-specific patterns of antibiotic usage (both with respect to the out-patient prescriptions as well as in agriculture and animal husbandry) [20], [21].

In this study we have performed a metagenome informatics based analysis to identify and profile the antibiotic resistance genes in the gut microbiomes of 275 individuals belonging to eight different geographical locations and age-groups.

## Methods

### Data Sets Used

Sequences of microbial genomic fragments (referred to as contigs) obtained from the gut microbiomes (also referred to as metagenome) of individuals belonging to diverse geographical locations were downloaded fromhttp://www.bork.embl.de/Docu/Arumugam_et_al_2011/downloads.html. These microbiomes/metagenomes, belonging to the guts of American, Danish, Spanish, French, Italian and Japanese individuals, were previously analyzed by Arumugam *et al*. [18]. Apart from these, genomic sequences obtained from the gut microbiomes of healthy and malnourished Indian children were also considered for this analysis [19]. Since the sequences obtained from the latter two microbiomes were available in comparatively smaller lengths (∼ 400 base pair), an additional step of obtaining longer length genomic contigs (as described in the next section) was specifically performed for the later two gut metagenomes. Gut metagenomic contigs corresponding to 116 European (Danish and Spanish) individuals, previously analyzed by Qin *et al* [22], were also downloaded from http://gutmeta.genomics.org.cn/. Apart from this, contigs corresponding to 90 gut metagenomes obtained from American individuals, sequenced as part of the Human Microbiome Project [23], were downloaded HMP-DACC website (http://www.hmpdacc.org/HMASM/). In addition, gut metagenomic datasets from 30 Chinese individuals, previously analyzed by Hu *et al* [21], were downloaded from the NCBI SRA database. Since the sequences in these metagenomic datasets were available as reads of lengths ∼ 150 bps, an additional step of contig assembly was performed on the Chinese gut metagenomes using MIRA [24]. The details of the different gut microbiomes used in the current study are provided in Table S1.

### Detection of Antibiotic Resistant Genes in Gut Microbiomes

#### (A) Gut metagenomes of American, Danish, French, Italian, Spanish, Japanese and Chinese individuals

The presence of homologs of various antibiotic resistance genes in these gut microbiomes was checked by performing BLASTx searches of the corresponding contigs against the ARDB database [25]. One of the key aspects of this step was to identify an appropriately stringent set of thresholds of BLAST parameters (e.g. identity and alignment length) to ensure (to the extent possible) that the homologs of the antibiotic resistance genes detected in the hitherto uncharacterized gut microbiota were functional (and did not correspond to distantly related non-functional homologs of these genes). At the same time, it was also important to ensure that the stringency of the thresholds did not result in high false-negative rates. Previous studies, characterizing functional antibiotic resistance genes from hitherto unknown organisms, have reported that the extent of identity between the sequences of a majority of such genes and their corresponding homologs in sequenced genomes was as low as 40–45% [12]–[14]. An identity threshold of 40% is also been provided as the default search criteria in the ARDB database. Similarly, a previous study attempting to identify antibiotic resistance genes in swine gut metagenomes (sequenced using the 454 sequencing platform) have used relaxed identity thresholds of as low as 35% and bit-score thresholds of greater than 60 [26].

In order to decrease the false-positive detection rate (and thereby increase the robustness of the predicted results), a sequence identity threshold (for the metagenomic contigs with the genes in ARDB) of greater than 50% was used in the current study. Subsequently, only those contigs, satisfying the above criteria and having an E-value <1e-8 with at least 90% of the length of resistance gene covered in the best-scoring alignment, were considered to have significant hits with genes in the ARDB.

#### (B) Gut metagenomes of healthy and malnourished children from India

Since the lengths of the sequences constituting the IN-CH-H and IN-CH-M metagenomes were around 400 base pairs (bp), longer length genomic contigs containing putative antibiotic resistance genes were obtained using the following steps.

Step 1*:* Sequences constituting the IN-CH-H and IN-CH-M metagenomes were compared with the genes in the ARDB database using a relatively relaxed E-value threshold of 1e-4. This step identified sequences as possible that showed even the remotest homology to any antibiotic resistance (AR) genes. The identified metagenomic sequences were then filtered using the following steps. The antibiotic resistance genes (having hits to these sequences), were analyzed by checking how much of their lengths were covered in alignment with the metagenomic sequences. Only those antibiotic resistance genes were considered which had at least 70% of their lengths being mapped by these sequences. Sequences mapping to these genes were retained for the next steps.

Step 2: For each shortlisted antibiotic resistance gene (as obtained in the previous step), the phylum level taxonomic affiliation of each metagenomic sequence mapping to it was obtained [19]. Based on these affiliations, the sequences were grouped into phylum level bins. Sequences assigned only at the level of Bacteria or Archaea were placed in separate bins.

Step 3: In this step, for each shortlisted antibiotic resistance gene (as in Step 1), a given phylum bin was first selected. Sequences belonging to this bin were then assembled using the CAP3 assembly program [27], along with the sequences belonging to the corresponding superkingdom bins (either Bacteria or Archaea). Sequences belonging to all other phylum bins were assembled separately one at a time, along with the unassembled sequences belonging to the corresponding superkingdom bins.

Step 4: Finally the antibiotic resistance genes encompassed by the contigs obtained above were profiled as described for the other gut metagenomes (refer to preceding section).

The steps 1–3 of this workflow are further summarized in Figure S1.

### Characterizing Antibiotic Resistance Genes in the Gut Microflora

#### Diversity and abundance of antibiotic resistance genes

For a given metagenome, the detected antibiotic resistance genes in each contig were tagged with a resistance profile (i.e. the antibiotic(s) to which the given gene products confer resistance to) of the corresponding antibiotic resistance gene in ARDB. The total number of resistance genes identified in the underlying gut microbial community was then obtained by collating the above information for the entire metagenome. Subsequently, the antibiotic resistance profiles corresponding to each of these genes was obtained.

The abundance of antibiotic resistance genes in each metagenome was calculated as the total number of antibiotic resistance genes detected per million base pairs of the corresponding metagenome. The gut microbiota of individuals exposed to a wide variety of antibiotic resistant organisms are expected to have resistance against a much larger array of antibiotics as compared to the gut microbiome of individuals having relatively lesser exposure. This is likely to be reflected in the overall diversity of antibiotic resistance in the corresponding microbiomes, i.e. the number of different antibiotics for which the resistance genes are detected per sequence volume of the corresponding metagenomes. Based on the premise, in this study, for each gut microbiome, the diversity of antibiotic resistance was also calculated as the number of antibiotics against which resistance genes were detected per million base pairs of sequence data.

#### Microbial groups harboring antibiotic resistance genes

For each metagenome, the probable microorganism(s) harboring each of the detected antibiotic resistance genes was identified using a strategy similar to that adopted by previous studies for accurate taxonomic classification of metagenomic sequences [18], [28]. For this purpose, the taxonomic assignment of each of the identified antibiotic resistance genes was obtained based on the taxonomic origin of the hit in ARDB and the degree of similarity (in terms of the sequence identity) between the hit and the detected antibiotic resistance gene as explained in Figure S2.

### Identification of Correlation Patterns among Antibiotic Resistance across the Gut Metagenomes

In order to identify groups of antibiotics having correlations in their abundance patterns across various metagenomes, the abundances of genes conferring resistance to each antibiotic were first obtained as follows. Given a metagenome, the number of detected genes conferring resistance to a given antibiotic was first counted. Subsequently, this number was divided by the size of the given metagenome in order to obtain the abundance of the given resistance profile (i.e. corresponding to the specific antibiotic) in the corresponding metagenome.

The abundances computed for a given antibiotic were then ranked across all the gut metagenomes. In order to remove the false positive patterns resulting due to sparse data, only those antibiotics resistance genes were considered which were detected in at least 10% of all the gut metagenomes. The ranked abundances of the resistances (corresponding to these antibiotics) were then plotted as hierarchical heat map using the R package. The heat map contained the antibiotics arranged on the vertical axis based on the similarities in the abundance patterns of the corresponding resistance genes.

### Identification of Gut Microbial Communities having Similar Antibiotic Resistance Profiles

The abundances of genes conferring resistance to each antibiotic across the metagenomes were first obtained. These abundance patterns were then subjected to a ‘Between Class Analysis’ (BCA), similar to that adopted by Arumugam *et al.* [18]. BCA is a special form of Principal Component Analysis (PCA), which first performs a pre-clustering of the data points. The centers of gravity of the obtained clusters are then used for computing the principal components. BCA was used in the current analysis since it is expected to be much more robust (to ‘noise’ within data points) than the conventional PCA approach. A detailed tutorial of the BCA approach as well as its application for the detection of Enterotypes, as used by Arumugam *et al*. [18], is available at http://enterotype.embl.de/enterotypes.html.

### Genes Facilitating Transfer of Antibiotic Resistance

Integrases and transposases are known to be implicated in the transfer of resistance genes across species [29], [30]. Detection of such genes in the neighborhood of the identified antibiotic resistant genes may indicate the presence of mobile elements, responsible for the transfer and dissemination of antibiotic resistance in the corresponding gut microbial communities. For this purpose, sequences corresponding to Integrase class 1 and IS transposase gene products were downloaded from the NCBI database (http://www.ncbi.nlm.nih.gov/). The contigs harboring the identified antibiotic resistance genes were subsequently subjected to a BLASTx search against the integrase and transposase sequences. The contigs showing significant similarities with these genes (alignment coverage >85% of the length for the integrase or transposase gene products, identity >90%) were identified [31]. In other words, this set of contigs harbored an integrase/transposase along with one or more antibiotic resistance genes. From this set, only those contigs containing an integrase/transposase gene in the neighborhood (within a distance of 10kb) of the detected antibiotic resistance gene were retained.

## Results

### Global Trend of Antibiotic Resistant Genes in Gut Microflora

**Figure 1:**
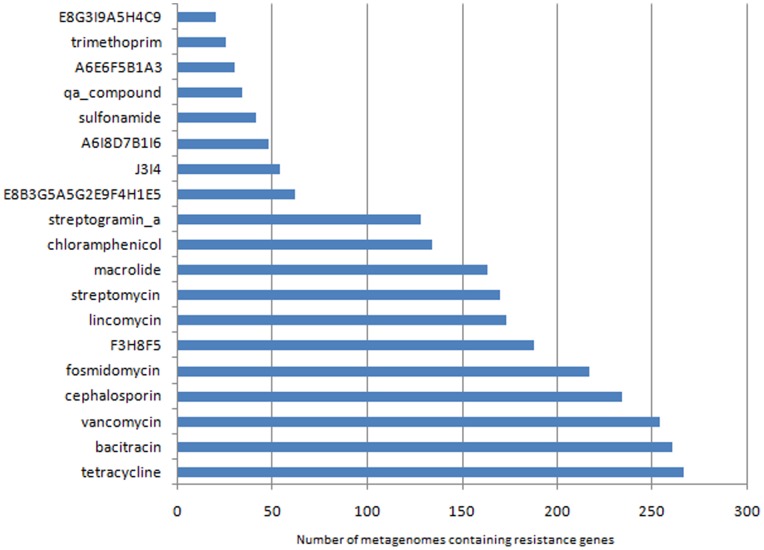
Number of gut metagenomes containing genes resistant to various antibiotics.

Homologs of genes conferring resistance to as many as 53 antibiotics were detected across the 275 gut metagenomes. Of these, resistance against tetracycline was observed to be the most common (present in 267 out of 275 gut microbiomes), followed by bacitracin (263 out of 275) and vancomycin (255 out of 275) (Figure 1). The probable taxonomic origins of the detected antibiotic resistance genes were also determined (Table S2). The genes conferring resistance to tetracycline were seen to primarily originate from the phylum Bacteroidetes (including the genera Bacteroides, Prevotella) and the family Clostridiaceae. In addition, results also indicate (Tables S2 and S3), a majority of genes conferring resistance to the poly−/glycopeptide-based antibiotic vancomycin and bacitracin could only be assigned at the level of phylum (i.e., the similarity observed between the identified genes with those present in the ARDB was relatively low). While the resistance against bacitracin (the genes *bcra* and *baca*) seemed to primarily originate from the phylum Firmicutes, followed by Bacteroidetes and Proteobacteria, the majority of genes conferring resistance to vancomycin (predominantly the genes *vanug*, *vang*, *vanrg*) were observed to belong to the phylum Firmicutes. These results indicate a predominance of antibiotic resistance genes originating from hitherto unknown organisms in the analyzed gut microbiomes.

Some of the identified antibiotic resistance genes in the analyzed gut microbial communities were observed to confer resistance to more than one antibiotic. These multi-antibiotic resistance profiles (referred here as wide-spectrum resistance profile) and the corresponding nomenclature codes used are listed in Table S4. Among them, genes having the profile F3H8F5 (conferring resistance to lincosamide, streptogramin B and macrolide) were detected in 182 out of the 275 microbiomes. Thus, apart from genes conferring resistance to single antibiotics, multi-antibiotic resistance genes were also observed to be present in the gut microflora across various nationalities.

### Geography-specific Patterns in the Overall Abundances of Antibiotic Resistance Genes in Adult Gut Microflora

**Figure 2:**
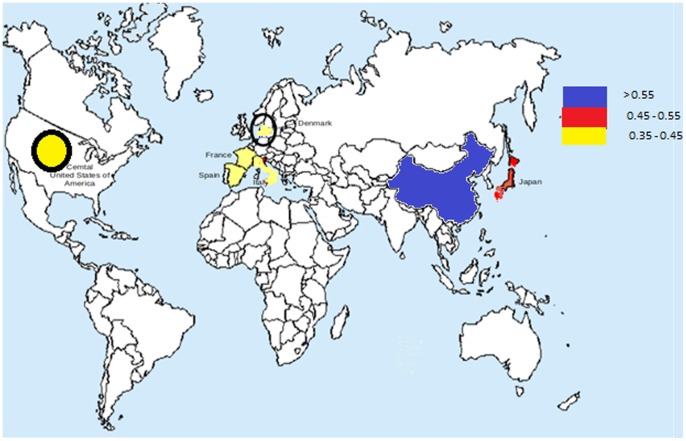
Abundances of antibiotic resistance (indicated in a color coded format) identified in the adult gut metagenomes from seven different nationalities.

**Figure 3:**
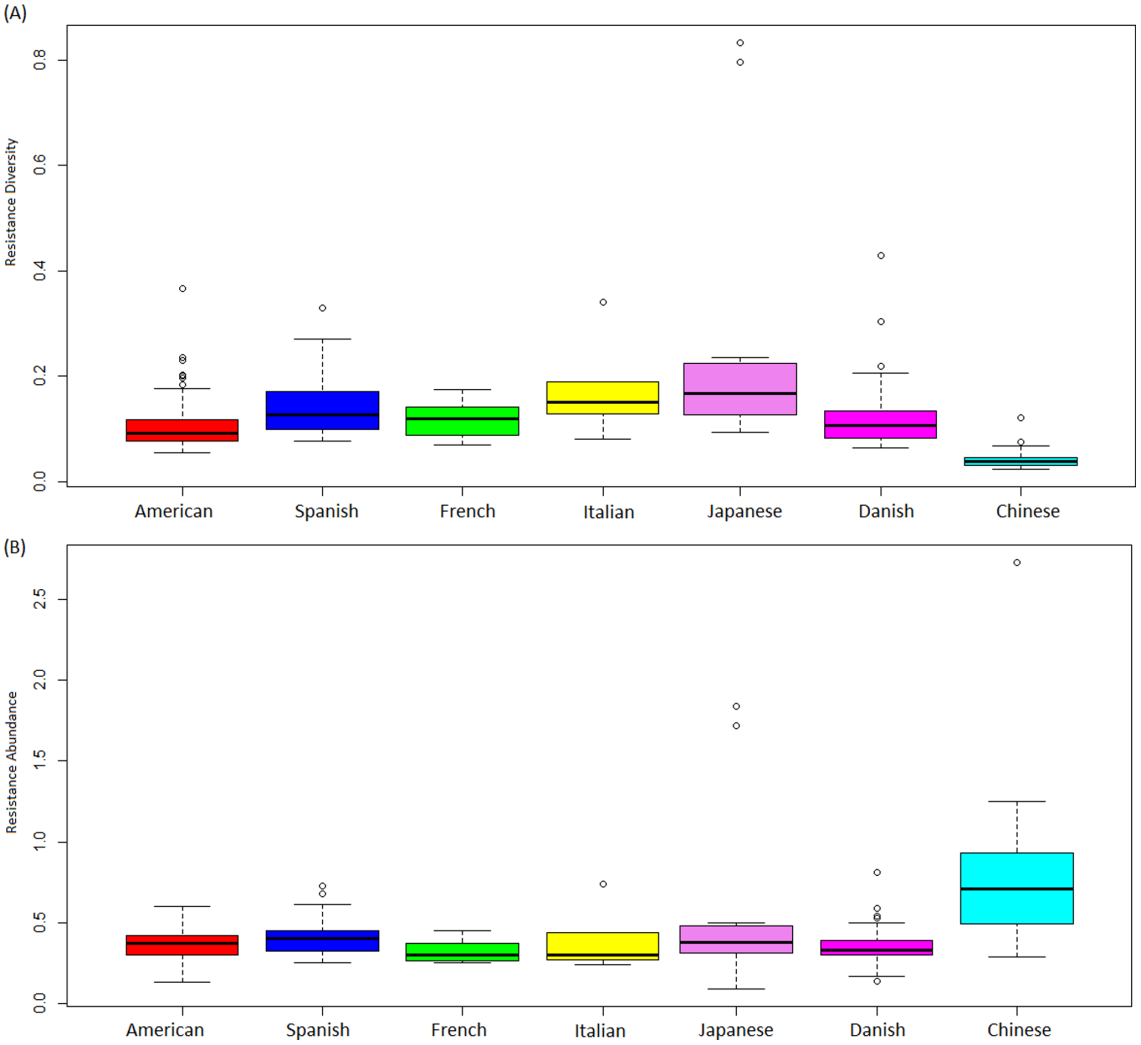
Diversity and Abundance of antibiotic resistance genes across nationalities. Diversity of antibiotic resistance genes across nationalities are shown in (A) and, the Abundances of antibiotic resistance genes, in the gut of individuals from various nationalities are shown in (B). Details of the gut microbiomes obtained from individuals belonging to various nationalities (indicated in the figure) are provided in Table S1.

Certain interesting patterns were observed when the diversities of the antibiotic resistance were compared across the gut microbiomes of adult individuals belonging to different nationalities (Table S5, Figure 2, Figure 3). The abundances of antibiotic resistance genes as well as the diversity of antibiotic resistance in the Southern European individuals (Spanish, French and Italian) were observed to be significantly higher as compared to the American and Danish individuals (two tailed t-test P<0.01). This indicated that the gut microbiota of Southern European populations have resistance to a wider array of antibiotics as compared to the American and Danish individuals. A similar trend was also observed by Forslund *et al*. [20]. Similarly, the diversity of antibiotic resistance as well as the abundances of resistance genes of the Japanese individuals was observed to be significantly higher as compared to the Danish and American populations (two tailed t-test P<0.02).

**Figure 4:**
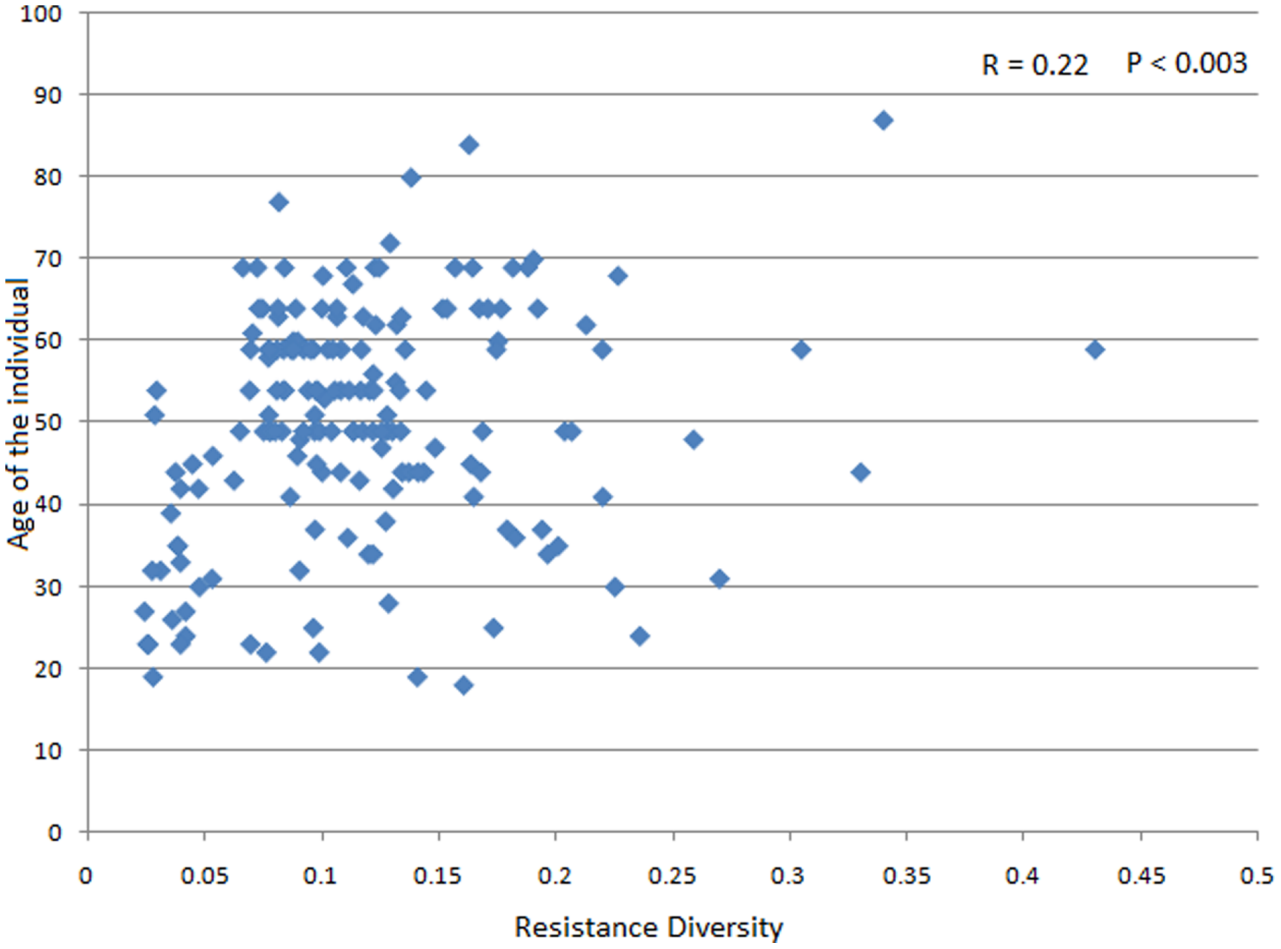
Variation of the antibiotic resistance diversity (in the 267 adult gut microbiomes) with the age of the individuals. Details on the names of the different gut microbiomes indicated in the figure are provided in Table S1.

The Chinese individuals were observed to have significantly higher abundances of antibiotic resistance genes as compared to those of other nationalities (two tailed t-test P<1e-12) (Figure 3). This result is also in line with those obtained by Hu *et al* [21]. However, the diversity of antibiotic resistance in the Chinese populations was observed to be significantly lower than those in other nationalities. The above results thus indicate that the Chinese individuals have higher number of genes conferring resistance to the same antibiotic as compared to those from other geographies. Within the 267 adult gut microbiomes, the diversity of antibiotic resistance was observed to have a rough correlation with the age of the individuals (R = 0.22, P<0.003) (Figure 4). This observation indicates that the reservoir of antibiotic resistance genes in the gut microbiome is likely to increase with the age of the individual.

### Geography Specific Antibiotic Resistance Patterns

Several antibiotic resistance genes were detected only in the Chinese population. Among the 157 antibiotic resistance genes whose homologs were detected in the current study, 35 were observed to be present only in the Chinese gut metagenomes (Table S6). These included the gene *bl2b_tem* (conferring resistance to cephalosporin and penicillin based antibiotics), *catb2* as well as *cata8* (both conferring resistance to chloramphenicol), *dfra12*, *dfra20* and *dfra21* (both conferring resistance to trimethoprim) and the multidrug resistance gene *mexw.* Another interesting observation was made with respect to the genes having the multi-antibiotic resistance profile F3H8F5. Although the resistance to the three antibiotics (lincosamide, macrolide and streptogramin B) corresponding to F3H8F5 was observed to be associated to the genes, *erma, ermb, ermc, ermf* and *ermg,* across a majority of the 275 gut microbiomes, the Chinese gut microbiomes were observed to harbour an additional set of genes (*lsa, oleb, srmb, msra* and *tlrc*) conferring resistance to the above three antibiotics A similar observation was also made for the genes conferring resistance to vancomycin as well as J3I4 (conferring resistance to vancomycin and teicoplanin). These results highlight that the Chinese gut microbiomes not only have a higher number of antibiotic resistance genes, but also harbour alternate resistance genes conferring resistance to the same antibiotic.

**Figure 5:**
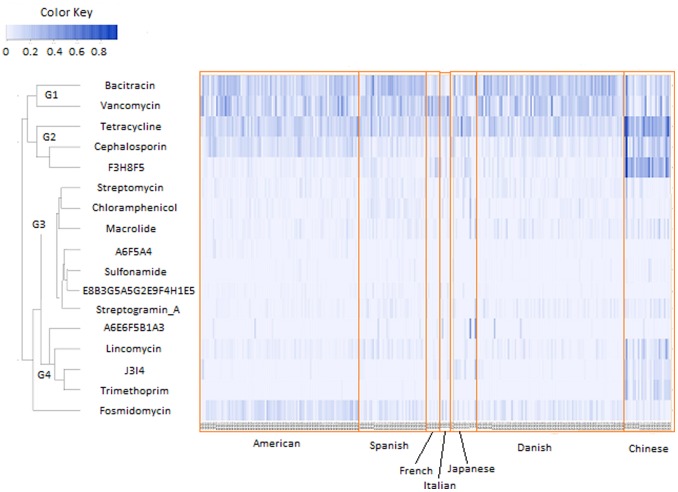
Variation of the abundances of genes conferring resistance to various antibiotics across the 267 adult gut microbiomes of seven nationalities. Only those antibiotics for which the resistance genes have been detected in at least 10% of the gut metagenomes are shown.

The resistance to the antibiotics cephalosporin and fosmidomycin were observed to be around two times higher in the American gut microbiomes as compared to those obtained from the European and Japanese geographies (T-test P-value <0.001) (Figure 5, Figure S3). The gut microbiomes of American individuals were observed to have lesser number of antibiotic resistance genes as compared to European and Japanese individuals. Similarly, resistance to chloramphenicol was observed to be more than two folds higher in the Spanish gut microbiomes as compared to those in the American/European/Japanese individuals (T-test P-value <0.007) (Figure 5, Figure S3). Similarly, the gut microbiota of four out of the six Italian individuals was observed to harbor resistance genes against aminoglycoside-based antibiotics. In contrast, genes conferring resistance to this antibiotic were detected in the gut microbial communities of only seven out of the 122 non-Italian European individuals. These results indicate the presence of country-specific patterns of antibiotic resistance within the European community.

### Comparison of Antibiotic Resistance Genes in the Gut Microflora of Children and Infants

#### Antibiotic resistance genes in the gut microbiomes of the Indian and Japanese children

**Figure 6:**
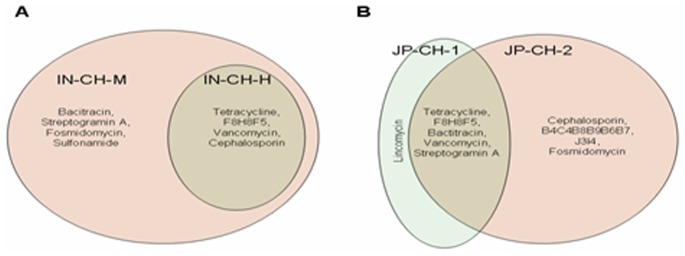
Comparison of identified antibiotic resistance genes in the gut microflora of (A) Malnourished Indian child (IN-CH-M) and Healthy Indian child (IN-CH-H) (B) Japanese children (JP-CH-1 and JP-CH-2).

The abundance of antibiotic resistance genes in the gut microflora of the two Japanese children was observed to be higher than that in their Indian counterparts (Table S5). It was also observed that the gut microbiome of the malnourished child from India harbored resistance genes against a higher number of antibiotics as compared to its healthy counterpart (Figure 6A). While the genes conferring resistance against tetracycline, cephalosporin, vancomycin and the wide-spectrum antibiotic resistance profile F8H8F5 were detected in the gut microbiota of Indian children (both healthy and malnourished), resistance genes against bacitracin, streptogramin A, fosmidomycin and sulfonamide were specifically detected in the gut microflora of the malnourished Indian child. These results indicated that the gut microflora of the malnourished child not only contained an over-abundance of pathogenic organisms reported earlier by Gupta *et al*. [19], but also had a larger reservoir of antibiotic resistance genes.

A comparative evaluation of the antibiotic resistance genes (Figure 6B) in the gut microflora obtained from the two Japanese children of the same family indicated that the gut microflora of JP-CH-2 (aged 18 months) had a higher abundance of antibiotic resistance genes as compared to its sibling JP-CH-1 (aged 3 years). The higher abundance of antibiotic resistance in the younger sibling is an unexpected observation.

#### Antibiotic resistance genes in the gut microbiome of Japanese infants

An extremely high abundance of antibiotic resistance genes was observed in the gut microflora of two Japanese infants (JP-IN-1 aged 4 months and JP-IN-4 aged 7 months). The gut microbial communities in these two infants were observed to harbor resistance genes against 21 different antibiotics (Tables S2 and S5). However, the microbial groups containing the genes resistant to these antibiotics were observed to be different in these two samples. While Klebsiella and Enterobacter genera accounted for most of the antibiotic resistance genes in the JP-IN-1 sample, a majority of these genes in the JP-IN-4 sample were observed to originate from the genus Escherichia and uncharacterized organism(s) belonging to Proteobacteria phylum. The over-abundance of antibiotic resistance genes in the gut microflora of these two infants is alarming.

### Correlated Antibiotic Resistance Patterns across Gut Metagenomes

Figure 5 depicts the ranked abundances of the resistance profiles corresponding to different antibiotic across the gut metagenomes. Based on the similarities in their resistance profiles, the antibiotics could be clustered into four groups, with resistance profile to fosmidomycin having abundance pattern distinct from the remaining four groups (Figure 5). The first group G1 contained the antibiotics vancomycin and bacitracin, the resistances to which were observed to be present across all gut microbiomes (but were relatively less abundant in the Chinese gut microbiomes). The group G2 contained the antibiotics F3H8F5 (conferring resistance to lincosamide, macrolide andstreptogramin B), tetracycline and cephalosporin. Resistance to these three antibiotics, although detected across all geographies, were observed to have a relatively higher abundance in the Chinese gut metagenomes. Amongst the non-Chinese gut microbiomes, while resistance to cephalosporin was observed to be higher in the American individuals, the resistance to F3H8H5 was observed to be higher in French/Italian/Japanese and certain Spanish individuals. The third group G3 contained the resistance profiles corresponding to macrolide, chloramphenicol, streptogramin A, streptomycin, sulfonamide, along with the multi-antibiotic resistance profiles of A6F5A4 (conferring resistance to aminoglycoside, macrolide and acriflavine) and E8B3G5A5G2E9F4H1E5 (conferring resistance to aminoglycoside based antibiotics isepamicin, butirosin, paromomycin, amikacin, neomycin, kanamycin, lividomycin, ribostamycin and gentamincin_b). The fourth group G4 contained the resistance profiles to lincomycin, trimethoprim, and J3I4 (conferring resistance to vancomycin and teichoplanin). Resistance genes corresponding to these three antibiotics were observed to have higher abundances in the Chinese gut metagenomes. The abundance pattern of fosmidomycin resistance followed an entirely different trend as compared to the groups G1–G4. As observed earlier, the abundances of the genes conferring resistance to formidomycin were observed to be higher in the American gut microbiomes and lowest in the Chinese gut microflora.

The observed correlation patterns are in line with the resistance potentials of the individual antibiotics across the different gut microbiomes reported by Forslund *et al*. [20]. A probable reason for the occurrence of such correlated resistance profiles could be the presence of specific organisms having specific resistance repertoires to the distinct sets of antibiotics. For example, a previous study identified the organism *Streptomyces coelicolor* to have drug efflux pumps specific for the antibiotics macrolides, streptogramins, and chloramphenicol (identified to belong to the group G3 in the study) [32]. Similarly, another study had identified several Enterococcus species containing genes conferring resistance to both tetracycline and F3H8H5 on the same plasmid [33]. In order to probe this aspect further, presence of multi-antibiotic resistance gene clusters occurring in the gut microbiomes were investigated.

### Multi-drug Resistant Gene Clusters in Gut Microflora

A total of 85 gene clusters containing multiple antibiotic resistance genes, located in close genomic proximity to each other, were also observed in the current study (Table S7). Interestingly, five different categories of such multi-antibiotic gene clusters were found to be prevalent across the different gut microbiomes. The first cateogory of such gene clusters harbored the *tetx* and/or *tetq* genes (known to confer resistance to tetracycline) and the *ermf* gene (known to confer resistance to the erythromycin class of antibiotics having the profile F3H8H5). Such multi-drug resistance gene clusters have been previously reported [34]. Interestingly, the genes conferring resistances to both tetracycline and F3H8F5 were found to have similar abundance patterns across all the gut microbiomes (group G2 in Figure 5). Furthermore, out of the 16 gene clusters containing tetracycline-F3H8F5-resistance genes, 11 were observed within the European population, indicating their higher prevalence within this geography. Moreover the genes identified in these gene clusters were observed to have more than 99% identity with the corresponding genes of *Bacteroides fragilis*, indicating the probable abundance of Bacteroides species similar to *B. fragilis* in the European population. The second category of gene cluster contained the genes *aph33ia/aph6id* and *sul3* conferring resistance to Streptomycin and Sulfonamide respectively. Similar gene clusters (containing resistance genes to Streptomycin and Sulfonamides) have been earlier reported in the pBP1 plasmid [35]. The pBP1 plasmid is known to be responsible for the transfer of sulphonamide resistance across bacteria [36]. Genes conferring resistance to these two antibiotics were observed to belong to the group G3 (Figure 5). A total of 10 such gene clusters were detected, out of which eight were specifically detected in the Spanish and Danish populations. Thus, the co-occurence patterns observed in the previous section correlate with the presence of multi-antibiotic gene clusters occurring in specific organisms inhabiting our gut. This further demonstrates that the analysis of the co-occurence patterns of various antibiotic resistance genes across gut microbiomes can be used to facilitate the detection of specific organisms harboring multi-antibiotic gene clusters.

The third category of gene clusters was detected specifically within the European populations and contained the *mexb* and *smed* genes conferring resistance to A6I8D7B1I6 (aminoglycoside, tigecycline, fluoroquinolone, beta_lactam, tetracycline) and fluoroquinolone respectively. In addition, the Chinese gut metagenomes specifically contained the remaining two categories of gene clusters. While the first category contained the genes *lnua* (resistance to Lincomycin) and *vanug* (conferring resistance to vancomycin), the second category contained the genes *lnua* and *mefa* (conferring resistance to macrolide) in close neighborhood.

### Identification of ‘Resistotypes’: Gut Microbial Communities having Similar Abundance Profiles of Antibiotic Resistance Genes

**Figure 7:**
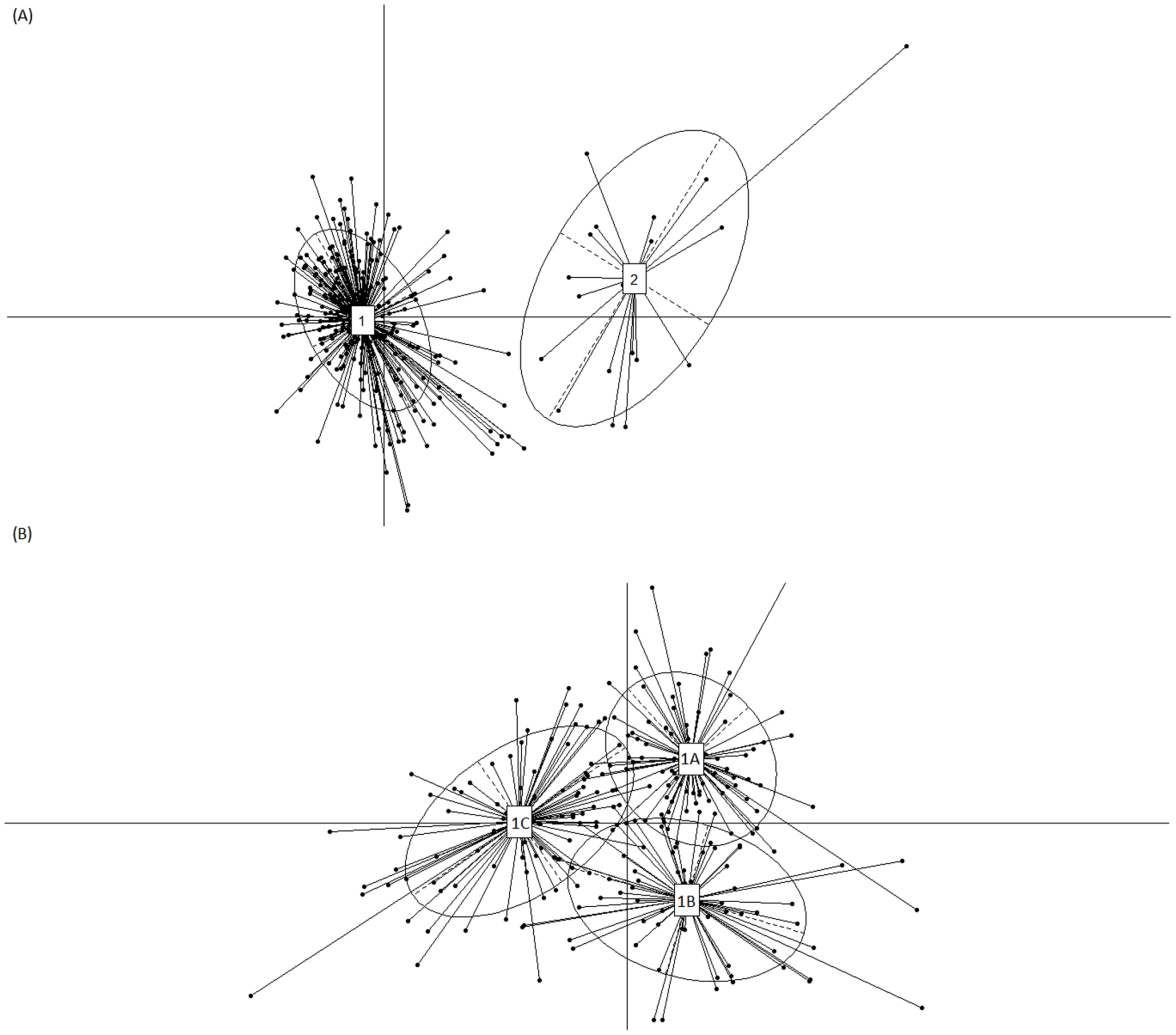
Distinct clustering of gut microbiomes based on similarities in the abundance patterns of genes conferring resistance to various antibiotics. Each cluster represents a ‘Resistotype’. (A) Resistotype 2 (observed specifically in Chinese individuals) and Resistotype 1 (containing gut microbiomes from individuals from all geographies). (B) Resistotype 1 resolved into three sub-clusters (Resistotypes 1A, 1B and 1C).

The BCA based clustering performed on the 267 adult gut metagenomes indicated two distinct clusters of gut microbial communities (Figure 7A). The two clusters represented groups of gut microbiomes having similar abundances of various antibiotic resistance genes. These two clusters were referred to as ‘Resistotypes 1 and 2’. While the Resistotype 2 contained 24 of the 30 Chinese individuals, the Resistotype 1 contained the gut microbiomes of the remaining 243 individuals. The Chinese-specific Resistotype 2 is a reflection of the distinct antibiotic resistance profile observed in the Chinese individuals (Figure 5). In order to further resolve gut microbiomes constituting the Resistotype 1, a second iteration of BCA was performed on the 243 individuals belonging to the Resistotype 1. This resulted in the resolution of the Resistotype 1 into three different sub-clusters, which were referred to as Resistotypes 1A, 1B and 1C (Figure 7B). The 267 adult gut microbiomes could therefore be associated with one of the four Resistotypes (Table S8).

**Figure 8:**
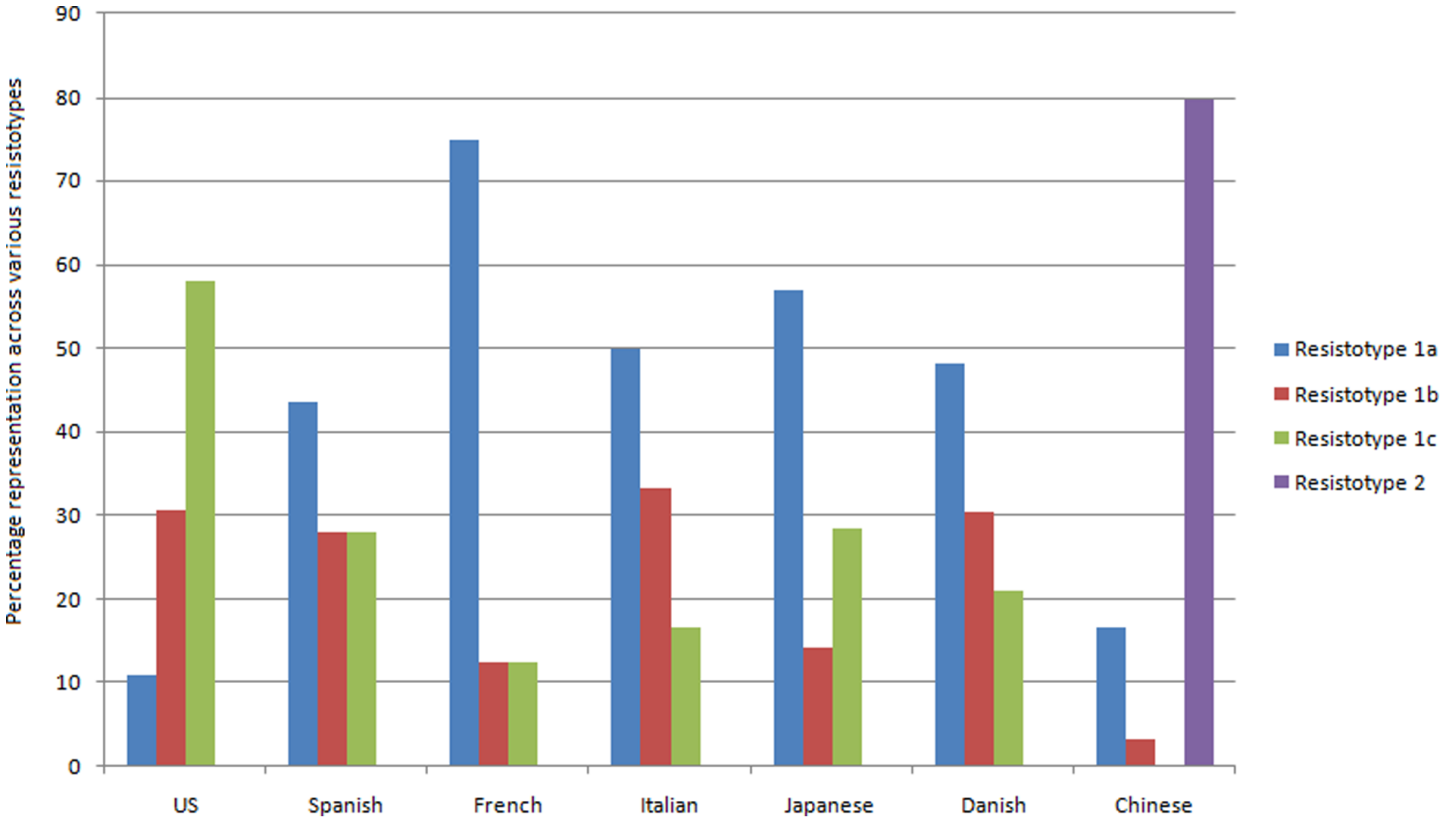
Percentage representation of gut microbiomes belonging to various nationalities within the four resistotypes.

Figure 8 shows the distribution of the adult gut microbiomes (from the seven different nationalities) in the four resistotypes. In contrast to the specific presence of Chinese individuals in Resistotype 2, each of the Resistotypes 1A, 1B and 1C was observed to contain individuals from all geographies. However, around 60% of American gut microbiomes were observed to be selectively placed in the Resistotype 1C, followed by Resistotypes 1B and 1A. Similarly, the gut microbiomes from the European and Japanese populations were observed to have a preference of Resistotype 1A over others. These results thus suggest geography specific preferences of gut microbiomes to belong to certain resistotypes.

**Figure 9:**
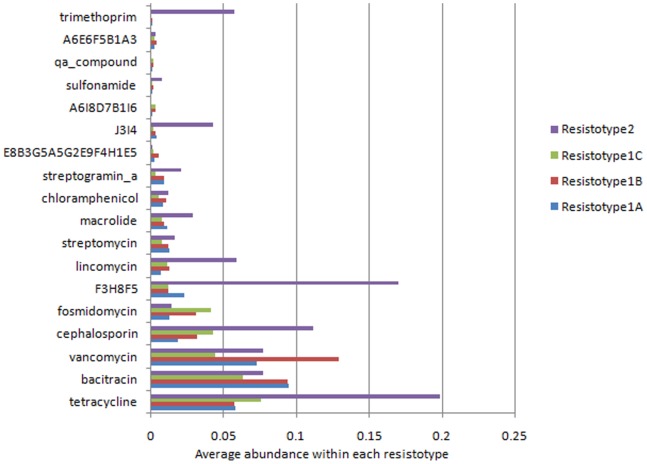
Abundances of the various antibiotic resistance profiles within the four different resistotypes.

Figure 9 depicts the abundances of the various antibiotic resistance profiles within the four different Resistotypes. Given the specific presence of Chinese individuals, the Resistotype 2 was characterized by a high abundance of genes conferring resistance to several antibiotics (similar to that observed in Figure 5). These included tetracycline, F3H8F5, cephalosporin, lincomycin, macrolide, J3I4 and trimethoprim. On the other hand, the Resistotype 1C, having a higher representation of the American individuals, was observed to contain a relatively higher abundance of genes conferring resistance to fosmidomycin and cephalosporin (as compared to Resistotype 1A and 1B). These results indicate that the composition of the resistotypes is indeed reflective of the underlying resistome corresponding to the constituent individuals.

### Elements Responsible for Transfer of Antibiotic Resistance Genes

**Figure 10:**
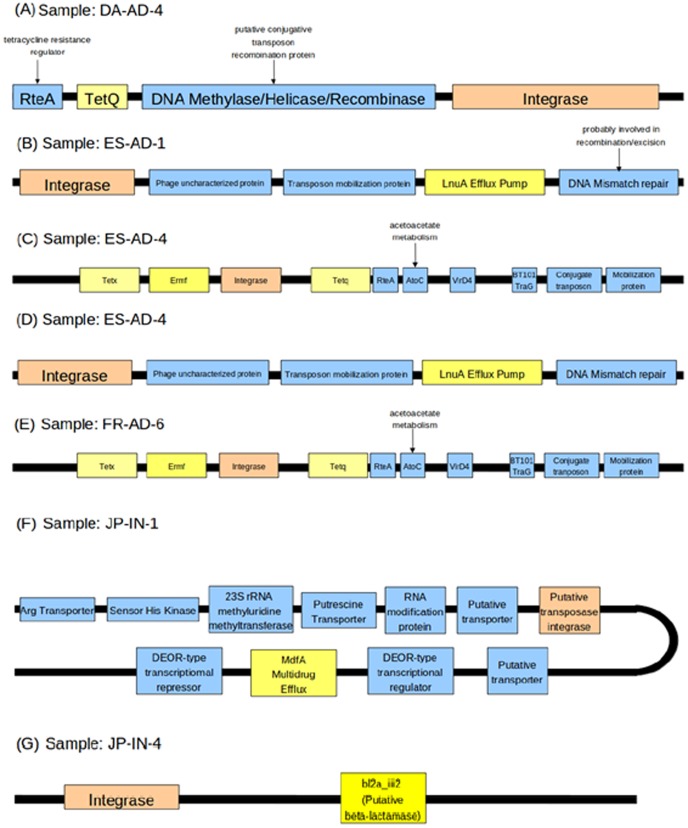
Details of the genomic contigs containing probable antibiotic resistance genes (highlighted in yellow) in the vicinity of integrase genes (highlighted in orange).

The current study identified seven distinct gene clusters, each containing an integrase and one or more antibiotic resistance genes on the same contig (Figure 10). Two of these identified clusters (in the ES-AD-4 and FR-AD-6 microbiomes) also contained the multi-drug resistant tetq/tetx/ermf gene clusters mentioned in the previous section. This observation further re-affirmed that these gene clusters may be part of conjugative tranposons or similar mobile elements responsible for the transfer of multiple resistance genes to other bacteria in the corresponding gut microbiomes. This observation is in line with previous studies wherein such clusters have been reported to be part of conjugate transposons that can be transferred across organisms. Two of these multi-antibiotic resistance gene clusters (in ES-AD-1 and ES-AD-4 samples) contained the *lnua* gene (known to confer resistance to lincomycin) with an integrase class 1 gene in its neighborhood. Interestingly, the closest hit to this gene in the ARDB was to the LnuA protein encoded in the plasmid of *Clostridium hemolyticus*. Previous studies have reported the existence of a similar transposon (referred to as the NBU2 transposon) in Bacteroides species that contain a related lincomycin resistance gene (*lin2*) in the neighborhood of integrase gene [31]. However, to the best of our knowledge, the occurrence of the mobile elements containing the *lnua* gene in conjunction with an integrase gene has not been reported in literature. To further investigate the above aspect, a BLAST search of this contig was performed against the transposon sequences in the NCBI non-redundant database. The closest hit of this contig was observed to be with the NBU2 transposon, with the integrase1 gene in a similar orientation as observed in the contig. Similarly the *lnua* gene was mapped to the region corresponding to *lin2* gene and a subsequent *mexa* gene in the vicinity of the *lin2* gene in the NBU2 transposon. This indicates that the detected transposable element is similar to the NBU2 transposon. Two regions detected in JP-IN-1 and JP-IN-4 microbiomes were observed to contain the beta-lactamase gene *bl2a_iii2* (conferring resistance to cephalosporin) and a gene encoding multidrug efflux pump (*mdfa*) respectively in conjunction with integrase genes.

## Discussion

A key advantage of the metagenomics approach is that it facilitates characterization of the functional properties of both the known as well as the hitherto uncultured/unknown organisms residing in a given environment. Concerted efforts by several independent groups as well as by consortia like Human Microbiome Project [23], [37] and the Meta-HIT project [18], [22] aim to sequence and comprehensively characterize metagenomes (sampled from different body sites) of large cohorts of human subjects (of diverse socio-economic and ethnic backgrounds). With the accumulation of metagenomic sequence data obtained from these projects, limitations with respect to sample size are expected to be addressed. The above aspect has been exploited in two previous studies to investigate the resistance potentials of gut microbial communities across individuals of diverse geographies [20], [21]. The current study presents a comprehensive investigation of antibiotic resistance genes present in the gut microbiomes of 275 individuals belonging to eight different geographies. Besides investigating the geography-specific trends in the resistance profiles, the current study also detects the existence of pairwise correlation patterns between the various antibiotic resistance profiles across gut microbiomes from different geographies. Notably, the detected correlation patterns also corroborate well with the resistance profiles of several multi-antibiotic resistant organisms reported earlier. The observed patterns are also reflected in the multi-antibiotic resistance gene clusters which were detected in the current study. A key observation pertains to the identification of specific multi-antibiotic resistance gene clusters, especially in populations of certain nationalities. Besides detecting the previously reported multi-drug resistant conjugate *text/ermf* tranposons and transferrable elements similar to pBP1 plasmid (specifically in the European populations), the study also detected *lnua* gene containing transposon (similar to the NBU2 transposon) in the Spanish gut metagenomes. Geography-specific identification of such transferrable elements is important since acquisition of resistance genes using such elements has been earlier reported to be responsible for several outbreaks. For example, acquisition of antibiotic resistance genes using SXT/R391 ICE family of transmissible elements shaped the pandemic spread of the seventh pandemic of *Vibrio cholerae* O1 in the early 1990s [38]. Likewise, a similar phenomenon has been observed for *Shigella dysenteriae* type 1 where acquisition of antibiotic resistance would spark epidemics. What triggers these epidemics when these families of antibiotic resistance genes are acquired is incompletely understood. Further studies focussing on the extensive identification of transferrable elements as well as the antibiotic resistant genes which they transfer are likely to provide insights into the above aspects.

Some of the geography-specific patterns of antibiotic resistance genes observed in the current study correlate well with earlier reports of self-medication and antibiotic usage across different countries. For example, the observed increase in antibiotic resistance in the Southern European populations clearly correlates with the earlier reports of higher level of self-medication and outpatient antibiotic usage in these countries [39], [40]. In addition, there is unequivocal evidence of a link between overuse of antibiotics in healthy livestock and drug-resistant disease in people. Furthermore, within the gut microbiomes obtained from the different European nationalities, certain country-specific patterns in antibiotic resistance are also observed. Another striking observation is the high abundance of antibiotic genes in the Chinese population, also reported earlier by Hu *et al* [21]. One of the key findings in the present study pertains to the detection of additional genes conferring to various antibiotics in the Chinese population. The country specific patterns of antibiotic resistance probably exist either due to the specific overuse of certain antibiotics in these countries or due to the presence of organisms harboring resistance against these specific antibiotics in the respective countries.

A key observation of the current study is the detection of ‘Resistotypes’, i.e. distinct groups of gut microbial communities characterized by presence of similar antibiotic resistance profiles. The use of the term ‘Resistotype’ is analogous to the term ‘Enterotypes’, which has been used earlier to denote individuals having similarities in their gut microbial community structure [18]. Furthermore, unlike in Enterotypes, the current study identified several geography specific trends related to the composition of the Resistotypes. The identification of the Resistotypes in the current study further indicates that individuals can be divided into groups based on the similarities in their gut resistome repertoire. Insights from such an analysis are expected to be of value in designing country-specific regulations on antibiotic usage.

The higher abundance of antibiotic resistance genes observed in the Japanese children as compared to the Indian children is probably due to the fact that developed countries have a longer history of antibiotic usage, leading to a higher resistance genes in the underlying gut microbiomes. Given the insufficient sample size (n = 2 for both the Japanese and Indian children), the above result may be sample-specific and may not convey a general picture. Nonetheless, previous studies, focusing on penicillin and vancomycin resistance patterns in the well-known pathogenic organisms *Streptococcus pneumoniae* and *Staphylococcus aureus,* respectively, had also indicated a similar picture wherein, abundance of resistance to certain antibiotics in Japanese individuals was observed to be higher as compared to their Indian counterparts [41], [42].

One of the observations of the current study pertains to the high abundance of antibiotic resistance genes in the gut microflora of the two Japanese infants. The original study reported that no antibiotics, probiotics and fermented food products were administered to these two infants at least four weeks prior to sampling [16]. Previous studies had speculated similarity of gut microbiota between mother and her infant [43]. Since, the gut metagenome of the mother (JP-AD-2) of one of the Japanese infants (JP-IN-1) was available; the probable taxonomic origins (microbes) of the antibiotic resistant genes in the maternal and infant gut microbiomes were compared. The results of the comparison of the antibiotic resistance genes in the gut microbial community of JP-IN-1 and JP-AD-2 indicated that although the resistance genes for tetracycline, streptomycin and macrolide were detected in the gut microbial communities of these two individuals, the microbial groups harboring these genes were observed to be different. For example, while the genes conferring tetracycline resistance were observed to originate from the family Vibrionaceae in the infant (JP-IN-1), such genes were seen to be harbored by bacteria belonging to the phylum Firmicutes, the genus Clostridium and the family Micrococcaceae, in the mother (JP-AD-2). Moreover, the gut microflora of the infant was observed to harbor resistance genes against a significantly higher number of antibiotics as compared to its mother (Table S5). These results indicate that the high abundance of antibiotic resistance genes in the Japanese infant (JP-IN-1) is not maternally acquired but is probably due to environmental exposure to antibiotic resistant organisms.

The findings of this study, although important, need to be comprehensively validated experimentally. It may be still too premature to decide whether antibiotics should be used in patients whose intestinal microbiota show multiple antibiotic resistance signatures. The significance of the presence of such antibiotic resistance signatures needs to be comprehensively evaluated. In cases like neonatal sepsis, e.g. by *Klebsiella*, the priority will be to get rid of the infection rather than withhold antibiotics because of the presence of sequence signatures of antibiotic resistance. These observations can definitely form the basis for further experimental validation and analysis that can be used in the efficient clinical management of bacterial infection using antibiotics.

Detection of such gene-operons was often limited due to the lower lengths of the contigs constituting the corresponding metagenomes. However, with the development of nano-pore based single molecule technologies, the lengths of the genomic sequences obtained during the sequencing process (as well as the assembled contigs) is likely to increase. The increase in length of the genomic contigs is likely to facilitate in the detection of entire antibiotic resistance gene operons (using metagenome informatics based studies), thereby increasing the prediction sensitivity of antibiotic resistance profiles.

## Supporting Information

Figure S1 **Graphical summary of the approach used for obtaining longer length contigs containing putative antibiotic resistance genes in the IN-CH-H and IN-CH-M metagenomes.** *Unassembled sequences refer to the sequences that have not formed contigs in the previous steps of the progressive workflow.
(PDF)

Figure S2 **Workflow adopted to obtain the taxonomic affiliations of the contigs showing significant similarity to the proteins in the Antibiotic Resistance Genes Database (ARDB).**
(PDF)

Figure S3 **Comparison of abundance of (A) Fosmidomycin resistance genes in American and Non-American Individuals, (B) Abundance of Cephalosporin resistance genes in American and European/Japanese (C) Chloramphenicol resistance genes in Spanish and American/Other European/Japanese individuals.**
(PDF)

Table S1 **Details of the gut metagenomes considered for the present analysis.**
(XLS)

Table S2 **Details of the antibiotic against which the resistance genes (and their taxonomic origin) in the gut metagenomic data sets obtained from the (A) American (B) Spanish (C) Danish (D) French (E) Italians (F) Indian (G) Japanese and (H) Chinese.** The numbers (in brackets) shown for each taxonomic group, indicate the number of contigs (in a given sample) that had the given antibiotic resistance profile and were assigned to the given taxonomic group (based on the alignment parameters).
(XLSX)

Table S3 **Detection of various antibiotic resistance genes in the analyzed gut metagenomes.** Resistance to multiple antibiotics are indicated by *.
(XLSX)

Table S4 **Nomenclature used for the identified multi-antibiotic resistance profiles along with the specific geographies where observed.**
(XLSX)

Table S5 **The normalized abundance of (A) Resistance diversity and, (B) Resistance genes, obtained for the 275 gut microbiomes.** * The tags AD, CH and IN: Samples are obtained from adult, children and infants respectively.
(XLSX)

Table S6 **List of resistance genes detected specifically in the chinese gut metagenomes.**
(XLSX)

Table S7 **Contigs showing hits to more than one antibiotic resistance gene.**
(XLSX)

Table S8 **Resistotype affiliations of the 267 adult individuals.**
(XLSX)
