# Supplementary material for: In Silico Analysis of Antibiotic Resistance Genes in the Gut Microflora of Individuals from Diverse Geographies and Age-Groups
Source: PLoS One. 2013 Dec 31;8(12):e83823. doi: 10.1371/journal.pone.0083823 (PMC3877126; doi:10.1371/journal.pone.0083823)
Supplement: Figure S1 — Graphical summary of the approach used for obtaining longer length contigs containing putative antibiotic resistance genes in the IN-CH-H and IN-CH-M metagenomes. *Unassembled sequences refer to the sequences that have not formed contigs in the previous steps of the progressive workflow. (PDF) [file pone.0083823.s001.pdf]

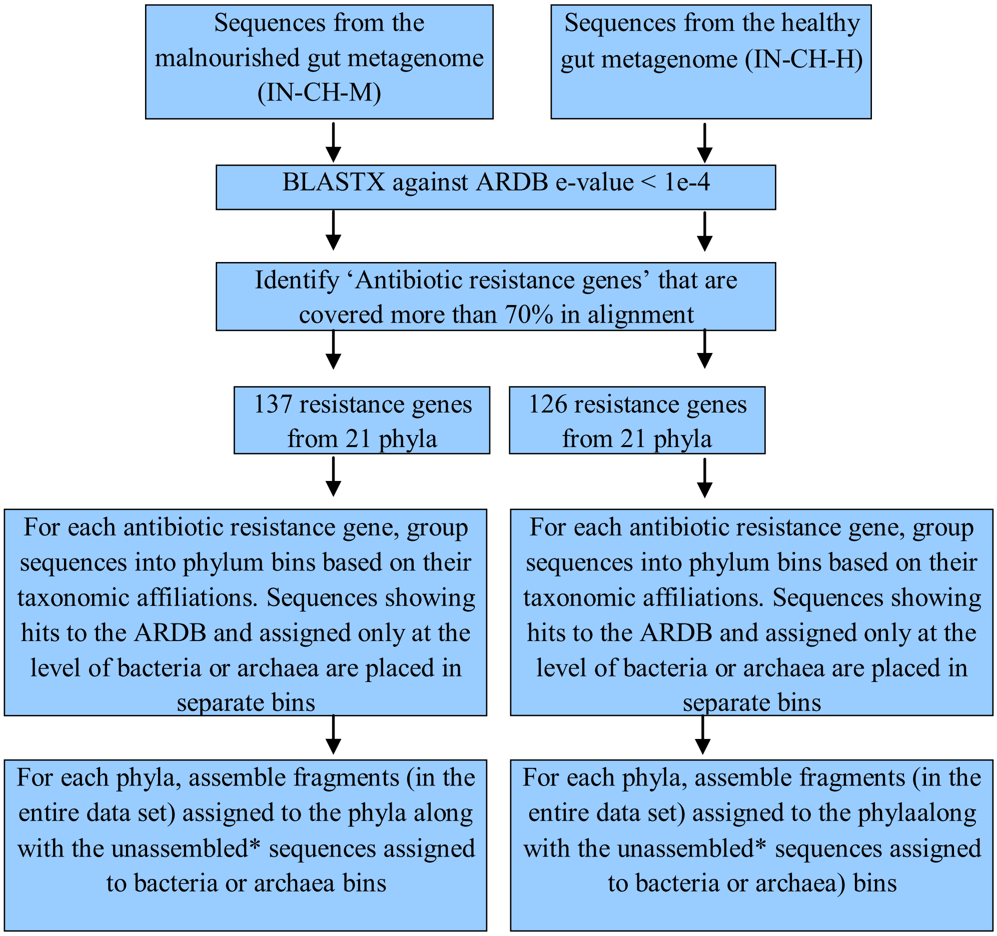

Supporting Figure S1: Graphical summary of the approach used for obtaining longer length contigs containing putative antibiotic resistance genes in the IN-CH-H and IN-CH-M metagenomes.

* Unassembled sequences refers to the sequences that have not formed contigs in the previous steps of the progressive workflow.
