# Supplementary material for: In Silico Analysis of Antibiotic Resistance Genes in the Gut Microflora of Individuals from Diverse Geographies and Age-Groups
Source: PLoS One. 2013 Dec 31;8(12):e83823. doi: 10.1371/journal.pone.0083823 (PMC3877126; doi:10.1371/journal.pone.0083823)
Supplement: Figure S2 — Workflow adopted to obtain the taxonomic affiliations of the contigs showing significant similarity to the proteins in the Antibiotic Resistance Genes Database (ARDB). (PDF) [file pone.0083823.s002.pdf]

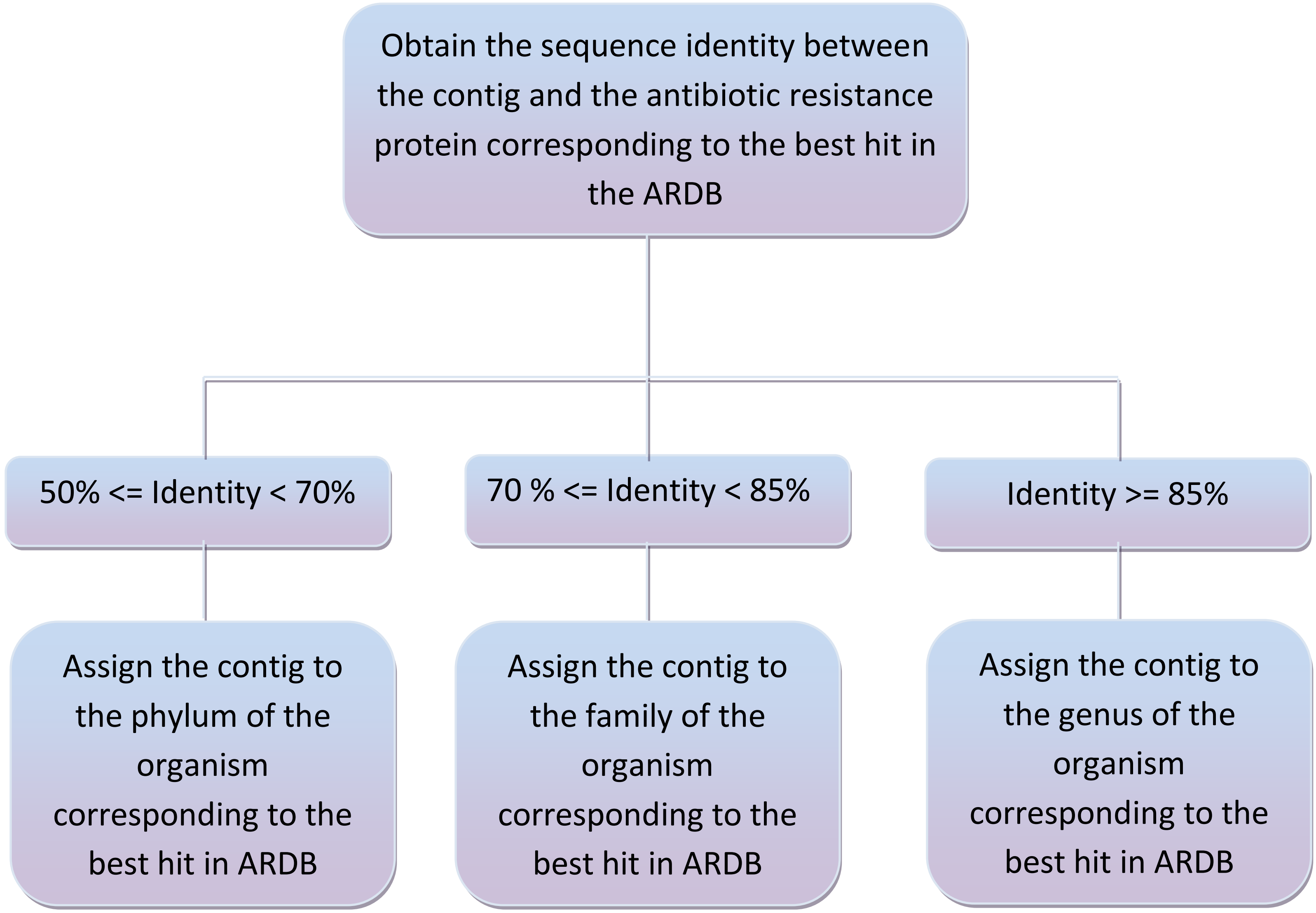

Supporting Figure S2: Worflow adopted to obtain the taxonomic affiliations of the contigs showing signficant similarity to the proteins in the Antibiotic Resistance Genes Database (ARDB).
