# Supplementary material for: In Silico Analysis of Antibiotic Resistance Genes in the Gut Microflora of Individuals from Diverse Geographies and Age-Groups
Source: PLoS One. 2013 Dec 31;8(12):e83823. doi: 10.1371/journal.pone.0083823 (PMC3877126; doi:10.1371/journal.pone.0083823)
Supplement: Figure S3 — Comparison of abundance of (A) Fosmidomycin resistance genes in American and Non-American Individuals, (B) Abundance of Cephalosporin resistance genes in American and European/Japanese (C) Chloramphenicol resistance genes in Spanish and American/Other European/Japanese individuals. (PDF) [file pone.0083823.s003.pdf]

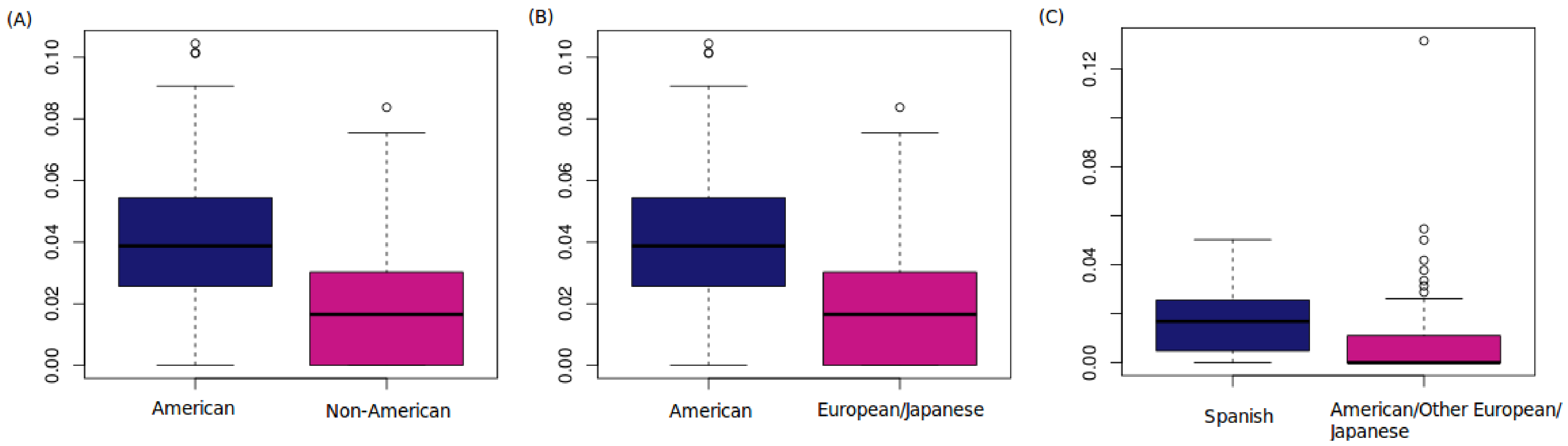

Supporting Figure S3: Comparison of abundance of (A) Fosmidomycin resistance genes in American and Non-American Individuals, (B) Abundance of Cephalosporin resistance genes in American and European/Japanese (C) Chloramphenicol resistance genes in Spanish and American/Other European/Japanese individuals
